# Understanding older adults’ acceptance of Chatbots in healthcare delivery: an extended UTAUT model

**DOI:** 10.3389/fpubh.2024.1435329

**Published:** 2024-11-19

**Authors:** Shulan Yu, Tianyue Chen

**Affiliations:** College of Furnishings and Industrial Design, Nanjing Forestry University, Nanjing, China

**Keywords:** Chatbot, older adults, UTAUT, perceived physical condition, self-actualization needs, technology anxiety, healthcare

## Abstract

**Background:**

Chatbots are increasingly integrated into the lives of older adults to assist with health and wellness tasks. This study aimed to understand the factors that enhance older adults’ acceptance of chatbots in healthcare delivery.

**Methods:**

This study proposed an extended Unified Theory of Acceptance and Use of Technology model (UTAUT), including aging factors of perceived physical condition, self-actualization needs, and technology anxiety. The model was tested by PLS (Partial Least Squares) with data collected from 428 Chinese citizens aged 60 and above.

**Results:**

The results reveal that performance expectancy, effort expectancy, and social influence significantly affected older adults’ behavioral intention to use chatbots. The facilitating conditions, self-actualization needs, and perceived physical condition significantly affected the actual use behavior of chatbots by older adults, whereas technology anxiety did not. Furthermore, the influence of effort expectancy and social influence on behavioral intention were moderated by experience.

**Conclusion:**

The behavioral intentions of older adults with low experience are more strongly influenced by social influences and effort expectancy. Furthermore, healthcare providers, designers, and policymakers should emphasize the impact of facilitating conditions, self-actualization needs, and perceived physical conditions on chatbot applications among older adults.

## 1 Introduction

Chatbots employ artificial intelligence (AI) and natural language processing (NLP) technologies to simulate human conversations, comprehend questions, and provide automated responses to inquiries (1). They have existed in commonly used smart devices such as smartphones, tablets, and smart speakers (2). Nowadays, they are extending into wearable technologies (3), including head wearables (4) and smartwatches (5), as well as multi-modal technologies (6, 7) and meta-universes (8). There is a growing demand for chatbots in the healthcare sector, with the objective of serving both patients and healthcare professionals. For instance, chatbots have become valuable tools for accessing real-time health information online (9). Chatbots can assist patients in determining whether their symptoms are transient or require further medical attention, offer health-enhancing advice, and encourage disease prevention measures (10). ChatGPT, a new generation of chatbots powered by advances in large-scale language modeling, can be utilized for various consultation, diagnosis, and educational tasks (11). When presented with typical “curbside consult” questions, patient presentations, or summaries of laboratory test results, GPT-4 generally provides functional responses that are useful for healthcare professionals in addressing patients’ concerns (12).

In light of the growing capabilities and accessibility of chatbots, their deployment in the healthcare sector offers a promising avenue for addressing specific needs across diverse demographic groups. One particularly significant group is older adults, who are facing increasing health challenges as they age. According to the United Nations Department of Economic and Social Affairs, the global population aged 65 and over was 761 million in 2021 and is projected to reach 1.6 billion by 2050 (United Nations, 2019). Furthermore, this aging population is contributing to an increase in the prevalence of chronic diseases globally, which is placing a significant economic burden on healthcare systems and society at large (13). Research shows that chatbots contribute to a better quality of life and overall health for older adults, thereby alleviating pressure on the healthcare system through their diverse functionality (14, 15). For example, chatbots can engage in activities such as singing, telling jokes, and conversations with older adults to help prevent dementia (16). By mimicking friendly conversations and asking one question at a time, chatbots facilitate personalized healthcare for older adults (17). In addition, their straightforward interactions encourage physical and mental well-being in older adults. A voice-based healthcare information chatbot, for instance, can assist in overcoming vision and dexterity barriers, enhancing medication awareness and adherence among older adults (18). The user-friendly chatbot interface also provides empathetic companionship for depressed patients over 65, potentially improving their social connectedness and reducing loneliness (19, 20).

Despite the benefits, older adults are often overlooked during the design and development stages of new technologies (21). This oversight can lead to usage challenges, as older adults may struggle with new systems and mistakenly attribute their difficulties to personal shortcomings rather than design flaws (22). Consequently, the older adult demographic often shows a more cautious adoption rate and a slower pace in becoming actively involved (23). The Unified Theory of Acceptance and Use of Technology (UTAUT) framework, which is derived from information technology, has been extensively used to examine the adoption behaviors of older adults. The UTAUT model has identified several factors that significantly influence older adults’ intentions to use medical apps (24), smartphones (25), online shopping (26), and social communication technologies (27). Previous research on the acceptance of chatbots has concentrated on the relationship between trust (28), anthropomorphic design (29), personal innovativeness (30), social self-efficacy (31), and use behavior in various domains, including higher education, finance, and digital marketing. Studies have also investigated the adoption of chatbots in a non-domain-specific context during the COVID-19 pandemic among Romanians (32) and identified key factors influencing the adoption of chatbots in healthcare literacy among students and younger adults (33). However, models used for younger users may not apply to older adults, who are the predominant users of healthcare services (34). This discrepancy arises because these groups value different aspects of technology (35). The specific factors that increase the adoption of chatbots in healthcare delivery among older adults remain unknown.

The promotion of chatbots in healthcare delivery for older adults can facilitate access to medical guidance and the management of their health, thereby reducing the burden and pressure on the social healthcare system caused by an aging population. Consequently, understanding the factors that influence older adults’ acceptance of chatbots in healthcare delivery is crucial. To this end, this study designs a modified UTAUT model that incorporates the aging characteristics of older adults, including perceived physical condition, self-actualization needs, and technology anxiety. The model is then subjected to empirical tests. The research questions are as follows: (1) What factors determine older adults’ behavioral intentions to use chatbots? (2) How do these factors facilitate or hinder older adults’ use of chatbots? (3) How does the moderating effect of experience influence older adults’ adoption? This study is expected to contribute to the existing literature on the subject by extending and empirically testing the UTAUT model with additional variables and providing valuable guidance to healthcare providers, designers, and policymakers.

## 2 Research framework and hypotheses development

### 2.1 Research framework

**Figure 1:**
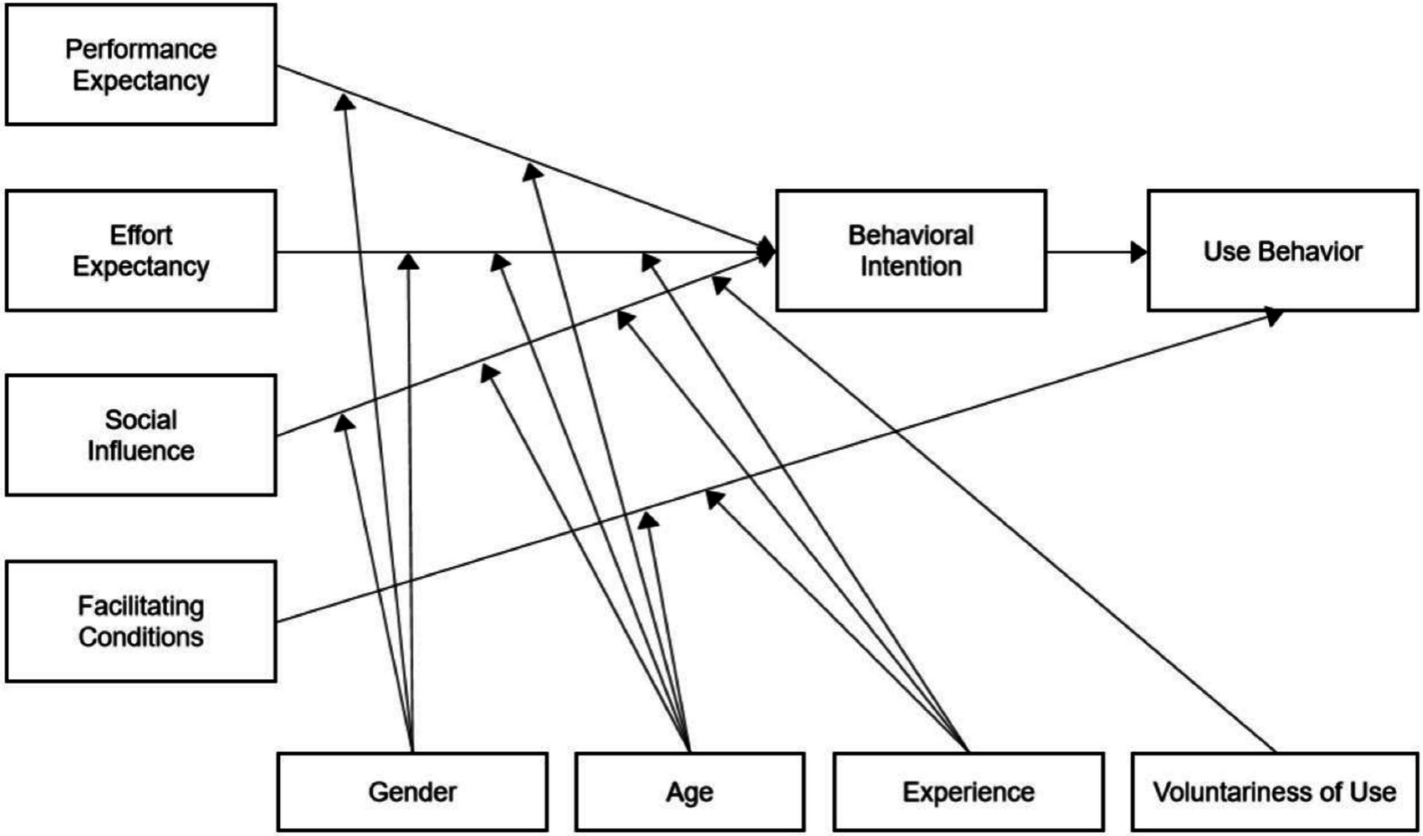
Unified theory of acceptance and use of technology (UTAUT).

Venkatesh and Davis combed through Technology Acceptance Model (36), Innovation Diffusion Theory (37), Theory of Reasoned Action (38, 39), Motivational Model (40), Theory of Planning Behavior (41), Combined TAM and TPB (42), Model of PC Utilization (43), and Social Cognitive Theory (44). Next, they integrated the above eight models to propose the Unified Theory of Acceptance and Use of Technology (UTAUT). This new model was able to explain 70% of the variance in usage intention, demonstrating a significant improvement over the original eight models and their extensions (45). It provides a helpful tool for understanding the drivers of acceptance (46). The model contains four main influences: performance expectancy, effort expectancy, social influences, and facilitating conditions. Gender, age, experience, and voluntariness are the moderators (47). Combining these constructs, UTAUT provides a comprehensive framework for understanding technology acceptance and use. The UTAUT model has been empirically tested in terms of the acceptance and use of chatbots in higher education (30), online health communities (48), academia (49), online shopping (50), and financial services (28). The choice of this model is explained by the fact that it has a higher predictive power and is more mature than the alternatives (33). The model has been widely used in research and practice to guide the design and implementation of technology interventions. By understanding these factors, this research can gain insights into what motivates older adults to adopt chatbots in healthcare and identify potential barriers to adoption. The UTAUT model is shown in Figure 1.

The UTAUT suggests that as the younger cohort matures, gender differences in how each perceives information technology may disappear (45). In addition, the behavior of the older adults in this study was voluntary to use chatbots in healthcare delivery. Therefore, the moderating variables of gender, age, and voluntariness were deleted. Experience has been presented as an important moderating variable in the UTAUT model (46). The effect of effort expectancy, social influences, and facilitating conditions tends to be more vital for those with lower experience levels (51). Older users with experience tend to be more independent and more likely to judge based on their perceptions (23). In a qualitative study of ChatGPT, experience was found to moderate the effects of factors in UTAUT on ChatGPT use (52). Therefore, we include the complete UTAUT as the baseline model and examine the moderating effect of experience in this quantitative study.

In addition, older adults’ adoption of technology is not purely technical but rather complex with multiple aspects (53). Aging is a distinguishing factor among older adults; therefore, older adults may have different adoption behaviors than younger users. Some prior research on technology acceptance behavior in older adults has developed older adult-specific trait structures to better understand older adults’ unique characteristics. Due to physical or psychological limitations, such as the gradual loss of sensorial capabilities of vision and hearing (54), older adults resist adopting new technologies. In addition, increasing the self-actualization needs of older adults can narrow the digital divide in technology-related opportunities (55). Some research found that technology anxiety is negatively associated with the perception and evaluation of smart health wearable devices and further affects the intention to use (56). The existing literature has examined the significant influences of aging factors on older adults’ adoption of medical software (57), online shopping (58), smart homes (54), and telemedicine (59).

**Figure 2:**
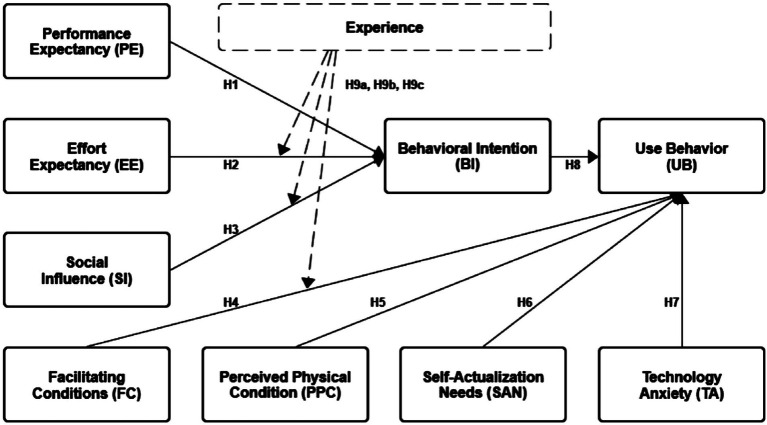
Research framework.

Venkatesh proposed that studies can adjust the original model by considering the user’s cognitive situation and incorporating new variables to expand and optimize it continuously (46). Therefore, we retained the original model of UTAUT and experience as moderating variables and included the aging characteristics of older adults as new variables in the research framework. The perceived physical condition, self-actualization needs, and technology anxiety are incorporated. The framework is shown in Figure 2, and the hypotheses are developed in the next section.

### 2.2 Hypotheses development

#### 2.2.1 Performance expectancy

Performance expectancy refers to the degree to which an individual expects technology to improve task performance (45). People use technology if they believe it will help them perform their job effectively (36). This study refers to the degree to which older adults believe that using chatbots in healthcare activities will help them perform their tasks more effectively and efficiently. Prior studies on chatbots suggest that performance expectancy is essential in predicting users’ behavioral intentions in various contexts (60). Camilleri and Menon et al. demonstrated that performance expectancy has a positive impact on the intention to use chatbots in education and the workplace (61). Similarly, Li et al. empirically revealed that the more performance expectancy, the more likely chatbots would be adopted in mental healthcare (62). However, the impact of performance expectancy among older adults’ behavioral intention to adopt chatbots in healthcare remains unknown. Therefore, the following hypothesis is postulated:

*H*1: Performance expectancy positively influences older adults’ behavioral intention to use chatbots.

#### 2.2.2 Effort expectancy

Effort expectancy refers to the degree of ease associated with using the technology (45). This study refers to the level of chatbot ease of use for older adults in healthcare delivery. Effort expectancy is a significant positive antecedent of chatbot use behavior (63, 64). Previous literature has documented the positive impact of effort expectancy on behavioral intention (65). For example, Mogaj et al. (2021) found that effort expectancy played a crucial role in adopting and using chatbots in the banking sector (66). Furthermore, in technology acceptance among older adults, effort expectancy has been identified as an essential factor that directly and positively influences older adults’ use of socially assistive robots (67), online shopping (26), digital health technologies (68), and mHealth (62). For older adults, chatbots that are easier to learn and operate are more likely to be adopted. Therefore, the following hypothesis is postulated:

*H*2: Effort expectancy positively influences older adults’ behavioral intention to use chatbots.

#### 2.2.3 Social influence

Social influence refers to how an individual perceives that others believe they should use a particular technology (45). The impact of social factors and the opinions of others on an individual’s technology adoption becomes significant when influential individuals or groups in a social network recommend a technology (69). This study refers to the degree to which older adults are influenced by the opinions of surrounding groups, such as friends, family, peers, and healthcare professionals, when choosing or using chatbots for healthcare services. Osta et al. found that social influence strongly impacted users’ intention to adopt AI conversational agents (chatbots) in the healthcare industry. Menon and Shilpa demonstrated that positive social influence can increase the user’s perception of the usefulness and ease of use of chatbots and reduce the barriers to adoption. Information and support others provide in the older adult’s immediate environment can significantly increase intentions to accept new technology (70). Therefore, the following hypothesis is proposed:

*H*3: Social influence positively influences older adults’ behavioral intention to use chatbots.

#### 2.2.4 Facilitating conditions

Venkatesh suggests that facilitating conditions are objective conditions in which individuals need to use information technology or products (45). This study refers to the degree to which older adults perceive that organizational and technological infrastructure exists to support using chatbots in healthcare. The significance of facilitating conditions has been highlighted in numerous studies regarding financial services (71), wearable devices (56), and telehealth (72). For chatbots, having laptops, smartphones, and the necessary knowledge and experts available for assistance increases the use behavior of chatbots in online health communities. When users receive more facilitating conditions to use chatbots, they will increase their intentions to use them (48). However, specifically for older adults, Yang et al. (2022) found that when older adults receive a smartphone training program, they are equipped with the resources and knowledge to use their smartphones (73). This resulted in no significant impact of facilitating conditions on the older adults’ use behavior of smartphones. Participants in this study were not systematically trained, and willingness to use chatbots in healthcare may increase when older adults perceive that the necessary organizational and technical support is available. Therefore, the following hypothesis is proposed:

*H*4: Facilitating conditions positively influence older adults’ use behavior of chatbots.

#### 2.2.5 Perceived physical condition

Aging involves biophysical and psychosocial changes that can increase challenges for older adults and potentially decrease their intention to use innovative technology. This study refers to the degree to which older adults feel that their physical condition limits their use of chatbots. Some studies have shown a negative correlation between the perceived physical condition and behavior (74). Chen et al. indicated that perceived physical condition can act as an internal control or inhibitory condition that affects the use of wearable devices by older adults (75). Similarly, Edelstein et al. argued that the underlying diseases induced by personal physiological conditions cause older adults more trouble using wearable devices (76). This trouble negatively impacted the adoption of the older adults’ use behavior of technology. For effective engagement with chatbots in healthcare, users need to have acceptable health conditions, including visual, auditory, and cognitive abilities. Disabilities like hearing or speech impairments may hinder effective interaction with technology among older adults (54). This study focuses on older adults aged 60 and above, who often face deteriorating health conditions that could impact their views on chatbot utility. Therefore, the following hypothesis is proposed:

*H*5: The perceived physical condition has a negative effect on older adults’ use behavior of chatbots.

#### 2.2.6 Self-actualization needs

The Self-actualization needs are at the highest level in Maslow’s hierarchy theory and represent an individual’s optimal potential and self-actualization (77). This study refers to the degree to which older adults perceive that their self-actualization needs are met through access to chatbots in healthcare. Previous research indicates that high self-actualization needs can facilitate the adoption of new technologies. Balakrishnan et al. further established a positive correlation between self-actualization needs and technology acceptance. In addition, previous studies found that self-actualization positively relates to older adults’ adoption of e-government services (78) and wearable health technology (79). Older adults will be more willing to use it if technology allows them to feel a sense of accomplishment, excitement, and happiness (80). The hypotheses about chatbots deserve further research. Therefore, the following hypothesis is proposed:

*H*6: Self-actualization needs positively influence older adults’ use behavior of chatbots.

#### 2.2.7 Technology anxiety

Technology anxiety is an aging factor associated with behavioral intention (81). It is a frustrating, emotional response to feeling worried or uneasy about accepting technology. Existing research has found that older adults experience technology-related anxiety, resistance, fear, and even technophobia when using digital devices or systems (82). In this study, technology anxiety refers to the extent to which older people feel intimidated or uncomfortable when they want to use chatbots. Studies by Hsieh et al. demonstrated that technology anxiety negatively impacts perceived ease of use and perceived benefits. This anxiety stems from a perceived lack of competence and can lead to rejection of technology. In the case of older adults, unfamiliarity and lack of confidence in technology use can reduce their expectations of performance and increase anticipated effort, thus impeding the acceptance and utilization of intelligent wearables (56). Consistent with these arguments, the following hypothesis is proposed:

*H*7: Technology anxiety has a negative effect on older adults’ use behavior of chatbots.

#### 2.2.8 Behavioral intention

Many theories related to technology acceptance, such as TAM and UTAUT, proposed the direct impact of the technology use intention on the actual behavior use (59, 83). For instance, Budhathoki et al. (2023) employed an adapted UTAUT framework to investigate university students’ adoption intention of ChatGPT in two higher education contexts– the UK and Nepal, suggesting that intention significantly influences actual usage. Chen et al. (2023) demonstrated that older adults intentions for wearable devices significantly impact their actual usage behavior. Therefore, we proposed a similar effect on older adults’ intention to adopt chatbots.

*H*8: Behavioral intention directly influences older adults’ actual use behavior of chatbots.

#### 2.2.9 Moderate effect

Users with different experience levels perceive effort expectancy, social influences, and facilitating conditions differently (45, 46). Current literature found that experience moderated the effects of these factors (57). Venkatesh et al. (2003) found that experience with technology can diminish the impact of perceived ease of use and perceived usefulness on adopting new technologies. This indicates that experienced users are more likely to make decisions about technology adoption based on their goals and needs rather than on external factors such as social influence or ease of use. Older users with high experience tend to be more independent and weaken the influences of effort expectancy, social influences, and facilitating conditions. A qualitative study showed that experience can moderate the impact of various factors on the use of ChatGPT, and individuals with more technology experience may find ChatGPT more user-friendly and helpful (52). Therefore, we proposed a similar effect in this quantitative study.

*H*9a: The influence of effort expectancy on behavioral intention will be moderated by experience.

*H*9b: The influence of social influence on behavioral intention will be moderated by experience.

*H*9c: The influence of facilitating conditions on use behavioral will be moderated by experience.

## 3 Methods

### 3.1 Measures

All of the scales were adapted and modified from previous studies. The main constructs of the UTAUT model (performance expectancy, effort expectancy, social influence, facilitating conditions, behavioral intention, and use behavior) were adopted from measurement constructs developed in related studies (45, 46). The perceived physical condition, self-actualization needs, and technology anxiety were each adapted from studies related to technology acceptance among older adults (74, 81, 84). Details on the questionnaire used and definitions of the constructs are shown in Table 1. All items were measured using the 5-point Likert scale, ranging from “strongly disagree” to “strongly agree.”

**Table 1:** Description and items of measurement constructs.

Constructs	Description	Items and contents
PE (45)	The degree to which older adults believe that using chatbots in healthcare activities will help them perform their tasks more effectively and efficiently	PE1: Chatbots in healthcare are beneficial. PE2: Some of the features of chatbots in healthcare have improved my life. PE3: It is convenient to use chatbots in healthcare.
EE (45)	The level of chatbot ease to use for older adults in healthcare delivery.	EE1: Learning to use chatbots to access healthcare was easy for me EE2: Using a chatbot is easy to understand and operate. EE3: It is easy for me to become proficient in using health chatbots.
SI (45)	The degree to which older adults are influenced by the opinions of surrounding groups	SI1: People around me think I should use chatbots in healthcare. SI2: Social and media encouragement and publicity made me want to use chatbots in healthcare. SI3: People around me support my use of chatbots in healthcare.
FC (45)	The degree to which older adults believe there is organizational and technological support for using chatbots in healthcare.	FC1: I have the necessary skills and knowledge to use chatbots in healthcare delivery. FC2: When I have a problem with chatbots in healthcare delivery, someone will help me. FC3: I have sufficient equipment resources, such as smartphones, the Internet, and computers, to support using chatbots in healthcare.
PPC (74)	The degree to which older adults feel their physical condition limits their use of chatbots.	PPC1: My current physical condition limits my daily activities. PPC2: I am hard of hearing, making using chatbots difficult. PPC3: I have a poor memory, making using chatbots somewhat tricky. PPC4: My eyesight is not so good, which makes it difficult for me to use chatbots in healthcare.
SAN (84)	The degree to which older adults perceive that their self-actualization needs are met through access to chatbots in healthcare	SAN1: Learning to use chatbots to access healthcare has increased my sense of fulfillment, satisfaction, and happiness. SAN2: Learning to use the chatbots gave me a sense of accomplishment.
TA (81)	The degree to which older adults feel intimidated or uncomfortable when they want to use chatbots.	TA1: Using chatbots in healthcare makes me nervous. TA2: Using chatbots in healthcare worries me.
BI (45)	The degree to which adults perceive that they intend to use chatbots for healthcare	BI1: I would like to use chatbots in healthcare. BI2: I am willing to learn to use chatbots to access healthcare. BI3: I want to use chatbots more in healthcare in the future.
UB (45)	The degree to which older adults actually use chatbots	UB1: I use chatbots a lot. UB2: I share with others the use of chatbots to access healthcare services UB3: I will continue to use chatbots in healthcare.

Moreover, this study contains three demographic information items: education, age, and gender. Education and age were scaled on a 5-point scale and coded as ordinal and interval variables. Consistent with previous research, gender was coded as a 0/1 dummy variable where 0 represented women (46). Experience was measured by the item, “How many months have you been using chatbots in healthcare delivery?” The scale options were: less than 2, 2–4, 4–6, 6–8, 8–10, and over 10 months. In this case, more than half a year of use is defined as a high-experience group, and less than half a year is defined as a low-experience group. The questionnaire was translated into Chinese to ensure that respondents were able to empathize with the content of the instrument and provide appropriate, meaningful responses (85). In addition, we conducted a pilot test with 98 respondents who were not included in the main survey. It showed that Cronbach’s alpha of all questionnaire items exceeded 0.7, proving that the questionnaire was reliable and usable (86).

### 3.2 Data collection

**Figure 3:**
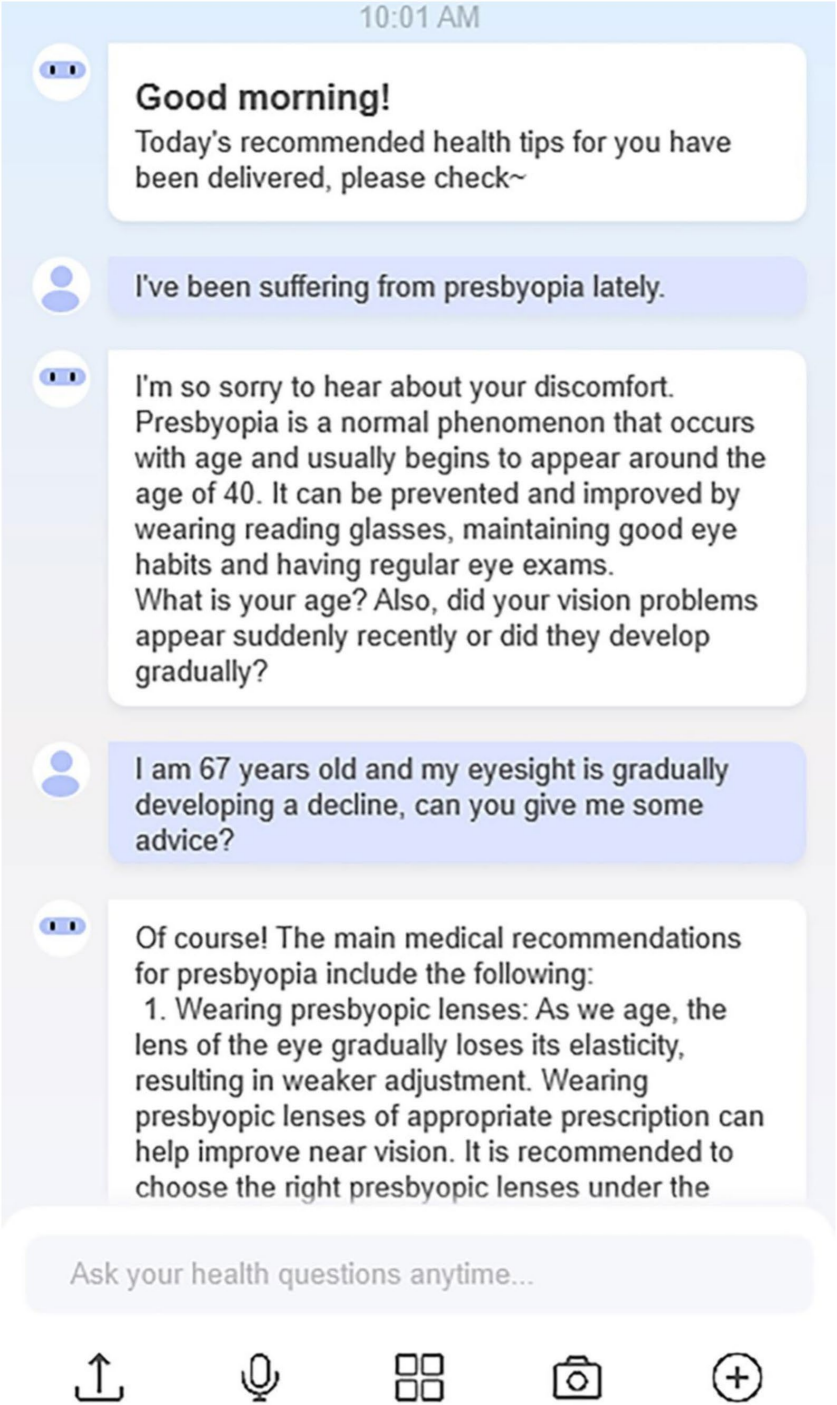
Chatbot interaction example.

Due to the lack of a reliable list of older adults with experience using chatbots to access healthcare services and their addresses, we used a convenience sampling method (87). Convenience sampling is a non-random sampling method that is cost-effective compared to other sampling methods and has been commonly used in technology acceptance studies (88). Based on information gathered from the convenient sample, an appropriate reference can be made for the population concerned (89). The survey was conducted on Questionnaire Star (Chinese online survey software) between December 2023 and April 2024. Electronic questionnaires were distributed among older adults in hospital outpatient clinics and wards and several healthcare services WeChat chat groups with a high concentration of older adults. Participants can start filling out the response questionnaire by scanning a QR code online or offline, or by clicking on a web link. In addition, snowball sampling was used in the data collection process to ensure that both the quantity and quality of responses were adequate. Participants were encouraged to share the link with eligible friends or family members. All participants were provided with a consent letter and an information sheet explaining the purpose of the study. To avoid the overclaim usage of the respondents, they were given flexible time to complete the questionnaire (90). Participants were also informed that they had the right to withdraw from participation during the study period, that their anonymity would be guaranteed, and that the results would only be reported in a summary format. To avoid potential bias, no gifts or incentives were given to the participants and all participated voluntarily. Furthermore, before the start of the survey, we gave an example of an interaction with a chatbot providing healthcare services (Figure 3). It was able to respond to users’ healthcare questions, give instant condition analysis, and recommend healthcare information.

Before the survey, two screening questions were used to ensure the accuracy of the data. The World Health Organization defines older adults as 60 years and above (91), we thus include this criterion. Specifically, we asked participants if they were 60 and above (yes/no). In addition, to reduce hypothetical answer bias among participants who had never used a chatbot in healthcare, we asked participants if they had ever used a chatbot in healthcare (yes/no). Participants who answered “yes” to both questions were considered eligible for this study. Participants who answered “no” were filtered out and did not continue with the survey.

**Figure 4:**
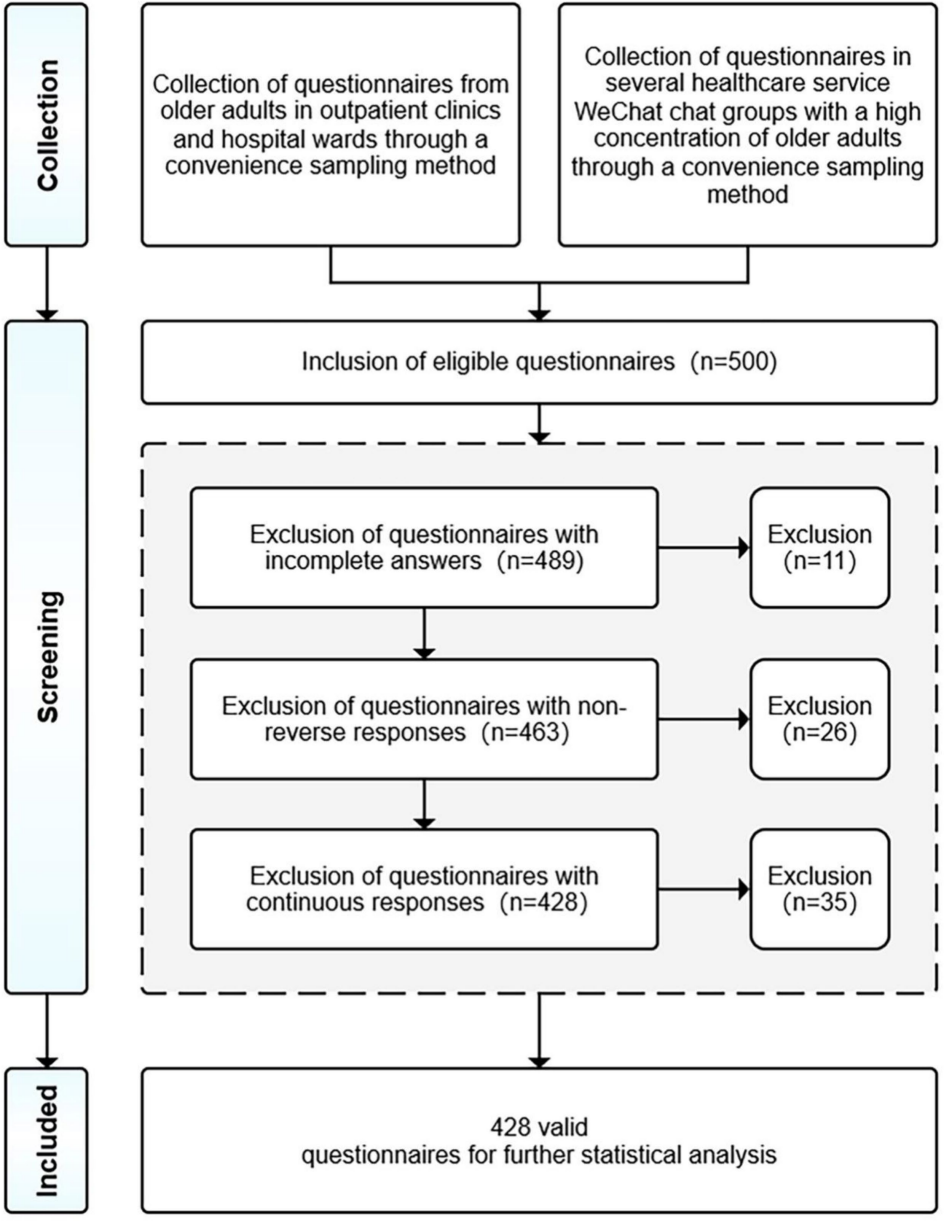
Data collection and screening.

Five hundred eligible questionnaires were collected. These questionnaires were subjected to three screening procedures (92). First, 11 respondents provided incomplete questionnaires and we removed them from the study. Second, the questionnaire contained a reverse-coded item, and respondents who provided non-reverse responses were considered to have responded carelessly, resulting in their questionnaire being excluded. Third, questionnaires that chose too many of the same option or showed some regularity in filling in the answers were also excluded. After rigorous screening, 72 invalid questionnaires were excluded, refer to Figure 4. Finally, 428 valid questionnaires were selected. According to previous research recommendations, the sample size should be 15–20 observations for each variable for generalisability purposes (93), and the total number of variables for the present study was 10, including moderating variables. Therefore, the sample size was considered adequate (>200).

**Figure 5:**
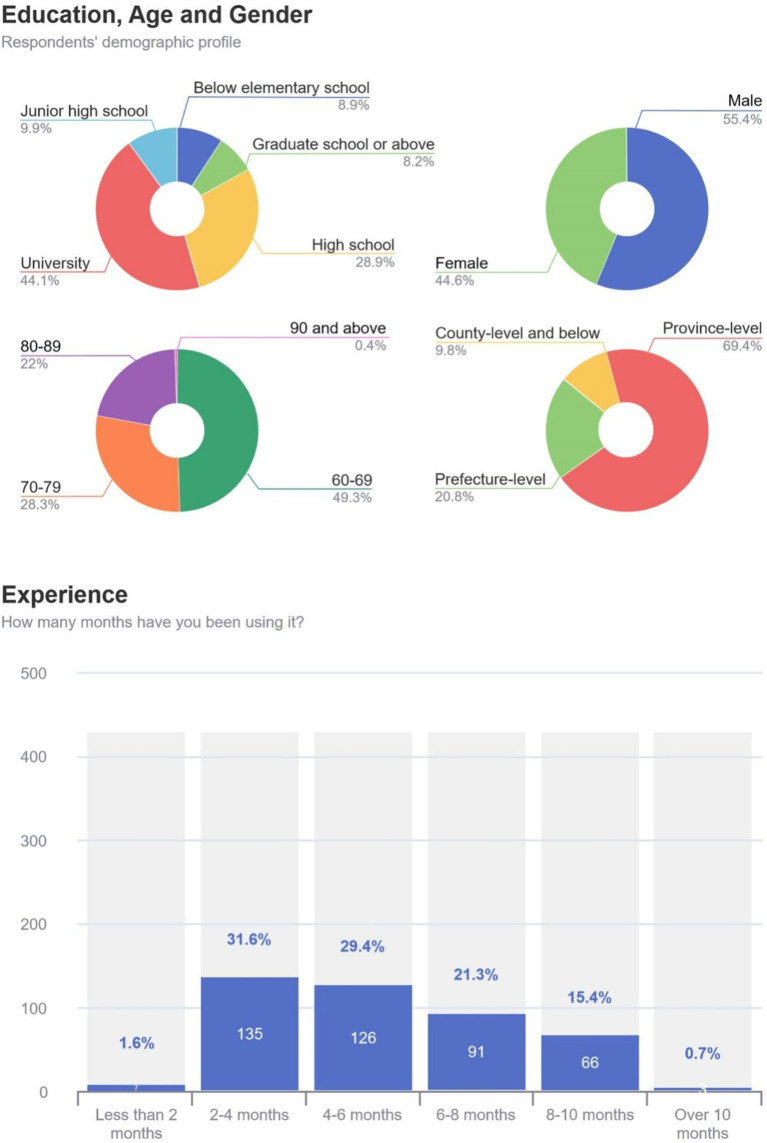
Demographics.

Figure 5 presents the respondents’ demographic profile, demonstrating that the university education majority is 44.1% and the high school education is 28.9%. Regarding age, 60–69 is the largest group, accounting for 49.3%. Regarding gender, males make up the majority at 55.4%, and female participants comprise 44.6%. Regarding the duration of use, 2–4 months is the largest group, accounting for 31.6%. The sample covered multiple administrative regions in China, with 69.4% in the province-level regions, 20.8% in the prefecture-level regions, and 9.8% in the county-level and below regions.

### 3.3 Data analysis

This study tested the hypothesized model using the partial least squares structural equation modeling (PLS-SEM), which is more common in information systems research. Unlike covariance-based structural equation modeling (CB-SEM), PLS-SEM employs a component-based analysis (94). PLS-SEM can maintain robust results in the face of small sample sizes and can maximize predictive validity (95). PLS-SEM explores multiple indicators that influence the dependent variable, explaining that a single PLS regression is particularly effective for data analysis (96). PLS-SEM is well suited for analyzing complex relationships between structures because it provides path coefficients and explains variance (97).

Data analysis consisted of three steps. First, a confirmatory factor analysis was conducted to determine the quality of the measurement model by assessing the reliability and validity of the construct. Second, blindfolding was used to test the robustness of the model, and bootstrapping was used to test the significance of the main effects. Third, bootstrap multigroup analysis and two-way ANOVA were used to determine the significance of the moderating effect.

## 4 Results

### 4.1 Measurement model

The high collinearity among indicators can produce unstable estimates and make it difficult to distinguish the distinct effects of individual manifest variables on the construct (98). Collinearity assessment involves computing each item’s variance inflation factor (VIF) by running a multiple regression of each indicator in the measurement model of the formatively measured construct on all other items of the same construct. As a rule of thumb, VIF values above 5 indicate collinearity among the indicators (99). In this study, all VIF values were less than 3, indicating that collinearity will not adversely affect future data analysis (refer to Table 2).

**Table 2:** Variance inflation factor.

	VIF
BI→UB	2.099
PE→BI	1.955
EE→BI	2.129
SI→BI	1.983
FC→UB	1.659
PPC→UB	1.526
SAN→UB	2.100
TA→UB	1.559

UB, Use behavior; Bl, Behavioral Intention; PE, Performance Expectancy; EE, Effort Expectancy; SI, Social Influence; FC, Facilitating Conditions; PPC, Perceived Physical Condition; SAN, Self-Actualization Needs; TA, Technology Anxiety.

Table 3 presents the results of indicator reliability, internal consistency reliability, and convergent validity measures. The rules of thumb for model evaluation are as follows: factor loadings should be 0.60 or higher to indicate good indicator reliability (100); composite reliability (CR) and Cronbach’s *α* should be above 0.70 to indicate adequate internal consistency reliability (101); and the average variance extracted (AVE) for each construct should be 0.50 or higher to demonstrate adequate convergent validity, meaning the construct explains more than 50% of the variance in the items (102). In this study, all factor loadings exceeded the 0.60 threshold. The Cronbach’s alphas ranged from 0.724 to 0.913, and the minimum composite reliability was 0.86, both above the 0.70 threshold. Additionally, all AVE values exceeded the 0.50 threshold. These results indicate that the constructs exhibit good indicator reliability, internal consistency reliability, and convergent validity.

**Table 3:** Reliability and average variance extracted (AVE) of the measurement model.

Constructs	Indicators	Factor loading	Cronbach’s alpha	CR	AVE
Performance expectancy	PE1 PE2 PE3	0.817 0.909 0.854	0.825	0.895	0.740
Effort expectancy	EE1 EE2 EE3	0.856 0.896 0.814	0.819	0.891	0.732
Social influence	SI1 SI2 SI3	0.762 0.865 0.910	0.802	0.884	0.719
Facilitating conditions	FC1 FC2 FC3	0.891 0.764 0.839	0.780	0.872	0.694
Perceived physical condition	PPC1 PPC2 PPC3 PPC4	0.915 0.828 0.913 0.884	0.913	0.936	0.785
Self-actualization needs	SAN1 SAN2	0.929 0.920	0.830	0.922	0.855
Technology anxiety	TA1 TA2	0.763 0.965	0.724	0.860	0.757
Behavioral intention	BI1 BI2 BI3	0.850 0.810 0.895	0.810	0.888	0.726
Use behavior	UB1 UB2 UB3	0.891 0.859 0.800	0.809	0.887	0.724

The correlations of the latent variables were measured by the AVE of each variable, as shown in Table 4. According to Fornell and Larcker, if the square root of the AVE is greater than the correlations among the constructs, the discriminant standards could be met (103). In this study, the square root of the AVE (shown diagonally in bold) exceeded the inter-construct correlations, indicating an appropriate level of discriminant validity. Therefore, all reliability and validity requirements were satisfied.

**Table 4:** Correlations of the latent variables.

	UB	BI	PE	EE	SI	FC	PPC	SAN	TA
UB	**0.851**								
BI	0.679	**0.852**							
PE	0.723	0.633	**0.861**						
EE	0.632	0.628	0.652	**0.856**					
SI	0.628	0.632	0.619	0.658	**0.848**				
FC	0.637	0.516	0.688	0.713	0.606	**0.833**			
PPC	−0.368	−0.352	−0.191	−0.314	−0.232	−0.287	**0.886**		
SAN	0.651	0.634	0.640	0.687	0.731	0.586	−0.179	**0.925**	
TA	−0.212	−0.339	−0.071	−0.129	−0.072	−0.133	0.539	−0.036	**0.870**

Bolded values on the diagonal are the square root of the average variance extracted (AVE) of the reflective scales. Values on the off-diagonal represent inter-construct correlations.

### 4.2 Structural model

The structural model evaluation for PLS-SEM requires the use of the explainable variance of the endogenous structure (R^2^) to describe the extent to which the independent variables of the current model explain the variation in the dependent variable; the calculation of Goodness of Fit (GoF) to describe the quality of the complete measurement model about the quality of the entire structural model; the Stone-Geisser’s Q^2^ to describe the predictive relevance of the model (94). R^2^ thresholds of 0.75, 0.50, and 0.25 are considered substantial, moderate, and weak, respectively (95). According to Wetzel et al., the cut-off values of GoF for small, medium, and large effect sizes are 0.10, 0.25, and 0.36, respectively. The Q^2^ values larger than zero indicate that the exogenous constructs have predictive relevance for the endogenous construct under consideration (104). In this study, the predictive power of behavioral intention (R^2^ = 0.523) and the use behavior (R^2^ = 0.614) was moderate, indicating that the model can explain a moderate degree of variance. The GoF value was calculated to be 0.510, which exceeded the 0.36 benchmark, indicating that the model has sufficient goodness of fit. The Q^2^ values of behavioral intention was 0.376 and use behavior was 0.437, indicating that the model has satisfactory predictive relevance for all the endogenous constructs. For a detailed list of R^2^, communality, and GoF, Q^2^, refer to Table 5.

**Table 5:** Robustness test.

	R^2^	Q^2^	Communality	GOF
PE	0.523 0.614	0.376 0.437	0.464	0.510
EE			0.451	
SI			0.432	
FC			0.386	
PPC			0.626	
SAN			0.475	
TA			0.292	
BI			0.574	
UB			0.436	

Hypotheses were tested by examining the magnitude of the standardized parameter estimates between the constructs and the corresponding *t-*values indicating the level of significance. When the t-value is greater than 1.96 and the *p*-value is smaller than 0.05, it is recommended to accept a hypothesis (105). Path analysis results are shown in Table 6. Bootstrapping was employed to evaluate the significance of the paths. In this study, technology anxiety had a non-significant effect on older adults’ behavioral intention to use chatbots (*t* = 0.552, *p* > 0.05), thus hypothesis H7 was not supported. The rest of the hypotheses were supported: Performance expectancy positively and significantly affected older adults’ behavioral intention (*t* = 7.144, *p* < 0.001), supporting H1. Effort expectancy positively and significantly affected older adults’ behavioral intention (*t* = 4.729, *p* < 0.001), supporting H2. Social influence positively and significantly affected the behavioral intention (*t* = 5.881, *p* < 0.001), supporting H3. The significance level of the facilitation conditions (*t* = 7.309, *p* < 0.001) was strongest in the UTAUT, which positively and significantly affected older adults’ use behavior, supporting H4. Overall, all four of the original hypotheses in UTAUT were supported. Furthermore, in the aging characteristics, perceived physical condition (*t* = 3.626, *p* < 0.001) and self-actualization needs (*t* = 5.276, *p* < 0.001) were positively associated with the usage behavior, supporting H5 and H6, respectively. Lastly, results also supported H8, which hypothesized that the behavioral intention is positively connected with the use behavior of chatbots (*t* = 8.051, *p* < 0.001).

**Table 6:** Results of hypothesis testing (H1–H8).

	Original sample (O)	Sample mean (M)	Standard deviation (STDEV)	T statistics (|O/STDEV|)	*p* values	Decision
H1	0.294	0.294	0.041	7.144	***	Supported
H2	0.248	0.249	0.052	4.729	***	Supported
H3	0.286	0.285	0.049	5.881	***	Supported
H4	0.281	0.280	0.038	7.309	***	Supported
H5	−0.136	−0.139	0.038	3.626	***	Supported
H6	0.251	0.250	0.048	5.276	***	Supported
H7	0.020	0.019	0.037	0.552	0.581	Unsupported
H8	0.333	0.333	0.041	8.051	***	Supported

Significance levels: *p < 0.05, **p < 0.01, ***p < 0.001.

### 4.3 Moderation effect of experience

After testing for main effects, we used bootstrap multigroup analysis to determine the significance of differences between the high-experience and low-experience groups. The result is significant at the 5% probability of error level if the *p*-value is smaller than 0.05 or larger than 0.95 for a certain difference of group-specific path coefficients (106). In this study, the differences in *p*-values are significant for effort expectancy (*p* < 0.05) and social influence (*p* < 0.05), as shown in Table 7. Therefore, H9a and H9b are supported. This suggests that the influence of social influence and effort expectancy on behavioral intention were moderated by experience. In addition, the effects of facilitating conditions on use behavior were significant in both the high-experience and low-experience groups. However, their difference in p-value was not significant. Therefore, hypothesis H9c is not supported. This suggests that the influence of facilitating conditions on use behavior was not moderated by experience and facilitating conditions are important drivers for both the high-experience and low-experience groups.

**Table 7:** Results of hypothesis testing (H9a–H9c).

	T statistics		Path coefficient difference	*p*-value difference	Decision
	High	Low			
H9a	0.476	6.065	−0.337	0.003	Supported
H9b	1.215	4.668	−0.421	0.001	Supported
H9c	6.244	9.481	0.056	0.487	Unsupported

**Figure 6:**
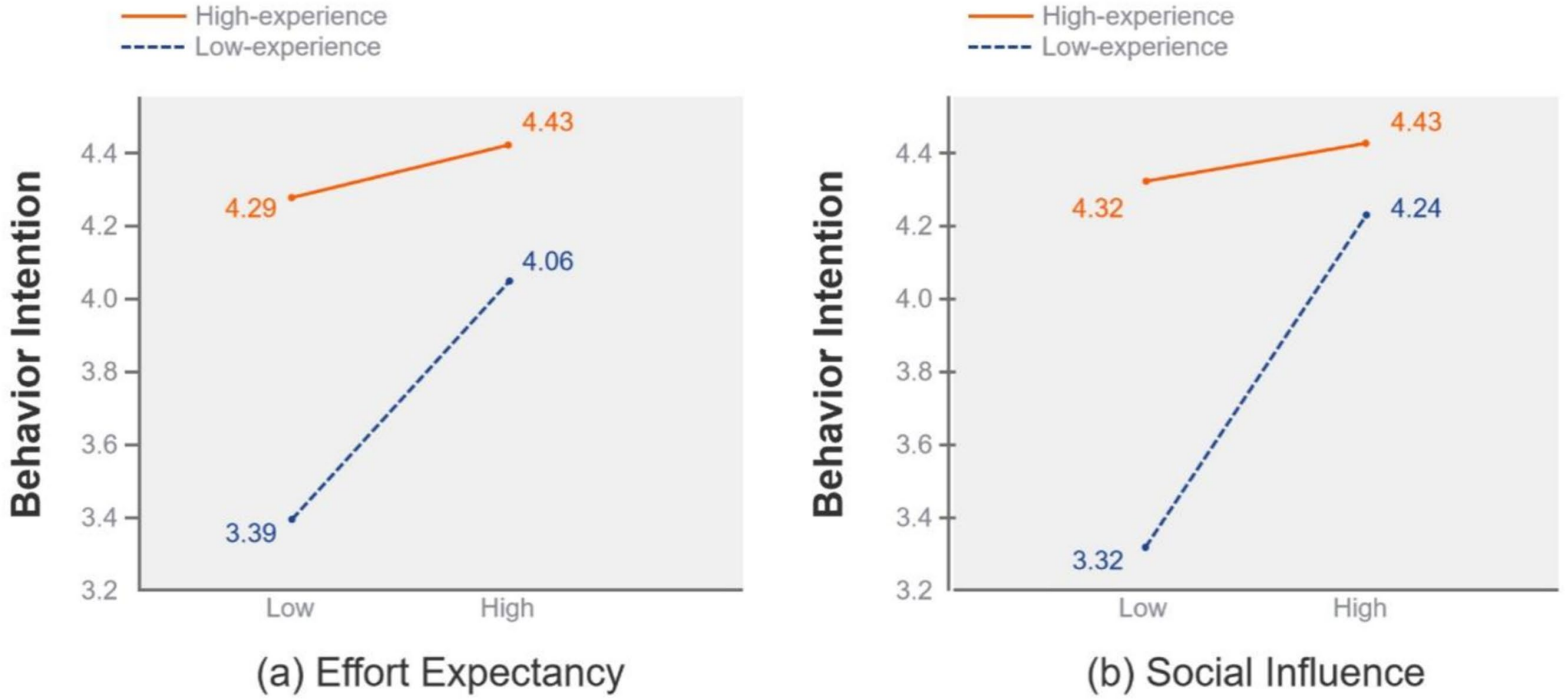
Moderation effect of experience (High vs. low).

The two-way ANOVA was used to compare the differences between the two groups further (107). The mean score splits the antecedents (effort expectancy, social influence, and facilitating conditions) into low and high groups. According to Hsu et al., the data were normally distributed with no significant outliers, making the mean split more robust. The results also showed that the influence of effort expectancy [*F*(1,424) = 11.568, *p* < 0.001] and social influence [F(1,424) = 34.131, *p* < 0.001] on behavioral intention were moderated by experience, which is plotted in Figure 6 respectively. The slope of the high-experience group is flatter than that of the low-experience group. Therefore, older adults with high experience are less affected by social influence and effort expectancy. Furthermore, the experience did not moderate the influence of facilitating conditions on use behavior [F(1,424) =1.87, *p* > 0.05].

## 5 Discussion

This study extends UTAUT by considering external variables to explore factors influencing older adults’ adoption of chatbots in healthcare delivery. The proposed model can explain 61.4% of the variance in use behavior (R^2^ = 0.614) and 52.3% in behavioral intention (R^2^ = 0.523). Seven of eight hypotheses were supported, including the four factors of the UTAUT and two aging factors. This study demonstrates that UTAUT theory can serve as a valid theoretical lens for exploring the factors influencing older adults’ intention to use chatbots in healthcare delivery. Furthermore, the study demonstrates the impact of experience differences on older adults’ behavioral intention to use chatbots in healthcare delivery. The theory and practical implications of the study’s findings were discussed.

### 5.1 Theoretical implications

In healthcare delivery, exploring the acceptance of chatbots has been the focus of a small number of empirical research. Nadarzynski et al. incorporated semi-structured interviews to explore the acceptability of chatbot systems for healthcare. Moldt et al. investigated the acceptance of medical students toward chatbots (108). Nevertheless, these survey responses were mainly drawn from students and internet users who are relatively experienced with digital technologies, particularly a young and educated cohort (21). The existing literature needs to look into the acceptance of older adults, the primary healthcare service users. (109). Consequently, this study has significant theoretical contributions to older adults’ acceptance of chatbots in healthcare delivery. This study developed and validated an expanded UTAUT model, including aging factors of perceived physical condition, self-actualization needs, and technology anxiety, to reveal the main factors that affect older adults’ intention and behavior to adopt chatbots in healthcare delivery. Furthermore, the study adds to the growing literature on experience and technology acceptance, with experience identified as moderate chatbot usage among older adults. The study presents the following insights.

First, the results suggest that performance expectancy and effort expectancy significantly influenced older adults’ behavioral intention to use chatbots in healthcare delivery. Consistent with previous studies, older adults want to avoid complex features and put in less effort and learning costs than younger people (110). This finding supports prior research. The ease of use and perceived usefulness are essential factors in technology adoption (63). Older users are more likely to adopt and integrate chatbots into their daily routines if the chatbot technology is easy to use and does not require much effort (52). Furthermore, social influence was a significant predictor of chatbot adoption among older adults in healthcare services. Consistent with the qualitative research on chatbots, older users may rely on recommendations from their peers (66). As older people are often not experts on many health-related issues, they are influenced by significant others in their social groups, such as their children, friends, and family members’ doctors (111). It is worth further noticing that facilitating conditions were the most important predictor of use behavior, consistent with previous studies (71). It is critical to have technical equipment and descriptive information to support older adults using chatbots in healthcare delivery.

Secondly, the results suggest that experience can moderate the impact of effort expectancy and social influence on older adults’ behavioral intention. Consistent with previous studies (52), low-experience users are more likely to put in less effort and learning costs, care more about technical support, and are more likely to listen to people. In contrast, older users with higher experience levels are less likely to be influenced by social media influence (23). This may be due to their greater reliance on their judgment and experience and their more nuanced understanding of the limitations and capabilities of the tool (57). Notably, the effect of facilitation conditions was stable and unmoderated. It further confirms that facilitation condition is an essential determinant in chatbot adoption for both older adults with high and low experience levels.

Finally, concerning the aging factors, the results suggest that the perceived physical condition is an obstacle to adults’ use behavior. Previous studies have found no negative correlation between the perceived physical condition and adoption due to the participants being physically healthy, and perceived physical condition by itself does not naturally lead to a better intention to adopt (54, 112). However, the participants of this study are older adults aged 60 years and above. Older adults’ hearing, vision, and cognitive abilities decline, so they become less sensitive to sound and take longer to find the right words to express themselves, hindering chatbot usage behavior. Furthermore, this study confirms that self-actualization needs are essential indicators of older adults’ use behavior. Previous studies have shown consistent results (113). Increasing the self-actualization needs of older adults can narrow the digital divide in technology-related opportunities and support (55). However, contrary to the assumption, technology anxiety did not impact adults’ use behavior. Previous studies have paid more attention to assistive technologies and health information technology, such as telehealth (50), wearable devices (69), and mobile health apps (114). Unlike these technologies, which require an operational threshold, chatbots use natural language interactions to deliver healthcare, reduce medication errors, and analyze health conditions (115, 116). The technology anxiety induced by chatbots could be alleviated due to natural, intuitive, and simple interactions (114).

### 5.2 Practical implications

This study provides practical insights for designing and implementing more aging-inclusive chatbots in healthcare. The use of chatbots in the field of older adults is still emerging, with a lack of specifically designed options for older users (109). The study suggests that designers should focus on enhancing the performance expectancy and effort expectancy of chatbots to promote older adults’ adoption of chatbots within the healthcare sector. For instance, many older adults cannot look at a picture and understand the meaning in front of minimalist icons designed for younger people (117). Thus, designers can address differences in icon comprehension related to participant age and design principles by adding text labels (118). In addition, the task-oriented interaction design is effective for less experienced older adults because it reduces the cost of effort and cognitive load for older adults (119). Meeting the needs of older adults through tailored and targeted approaches is essential, which could reduce the effort required for them to seek information independently, thereby increasing their use of chatbots in healthcare services. For instance, developers can use the geographic location of older adults to push nearby hospitals to them (120), automatically recommending relevant medical services based on personalized information (121, 122).

Moreover, policymakers should focus mainly on facilitating conditions and social influence and tailor their implementation strategies to ensure maximum reach and adoption of the chatbots (52). Policymakers should emphasize the provision and construction of infrastructure and expand more equipment for older adults (123). In addition, chatbot education and training programs in healthcare should be structured so that older adults receive the guidance they need at the right level of experience. For example, older adults with lower levels of experience are more susceptible to the positive effects of social influences, so training can be structured through creating interest and learning groups by focusing on groups with similar learning experiences (25). Intergenerational cooperation programs can also be developed to allow older adults to receive support from their children.

Finally, the perceived physical condition is a primary barrier to technology acceptance among older adults, and healthy conditions serve as a precondition for older adults’ adoption of chatbots (75). The multimodal interactions of chatbots can compensate to some extent for sensory or cognitive impairments and mitigate the negative impact of the perceived physical condition on older adults. For example, voice input commands can address, to some extent, the problem of finger inflexibility in older adults (124). The depression and Alzheimer’s screening tool Alexa requires only a yes or no answer to the questions to complete the assessment (125). Refer older adults for medical advice and health management services by uploading a picture of a disease condition or a medical report (126, 127). Therefore, healthcare providers can integrate chatbots into existing systems to provide multimodal interaction capabilities. Specifically, chatbots can be compatible with assistive devices such as hearing aids and vital signs monitors (128), providing voice input (129) and screen-reading capabilities (130), thereby countering the barriers posed by declining physical condition and increasing the use of chatbots among older adults.

### 5.3 Limitations and future research

Despite the theoretical and practical contributions of this study, there are limitations. First, while convenience sampling allows us to collect data from different regions of China, this approach may need to be more represented. Future studies should consider more systematic sampling methods, such as stratified random sampling or whole cluster sampling, to improve the representability of the sample. Second, most of the samples collected for this study were from urban areas in China. This may be because older adults in urban areas are more likely to have experience with chatbot healthcare technology than those in rural areas. For the findings to have potential for generalization, future surveys should consider a better representation of the population across all samples of demographic variables. The proportion of rural older adults can be increased by providing them with equipment, technical support, and training for the first use of chatbots. Finally, technology adoption among older adults is complex and multifaceted. Future research should consider the impact of different influencing factors, such as investigating the perceived privacy risks and perceived psychological risks of older adults in accepting and adopting chatbots.

## 6 Conclusion

This study extends the original UTAUT model with new variables from the perspective of older adult characteristics. It demonstrates that UTAUT theory can serve as a valid theoretical lens for understanding older adults’ acceptance of chatbots in healthcare delivery. The results highlight that facilitating conditions, perceived physical conditions, and self-actualization needs play important roles in older adults’ use behavior. Furthermore, this study tests the moderating effect of experience on technology adoption among older adults. Older adults with lower experience levels were more concerned about ease of use and social support. Understanding the factors that motivate older adults to use chatbots in healthcare delivery can offer valuable guidance for developing inclusive policies and designing age-friendly chatbots in healthcare delivery.

## Data Availability

The original contributions presented in the study are included in the article/Supplementary material, further inquiries can be directed to the corresponding author.
